# Effect of Pharmacological Interventions on the Fronto-Cingulo-Parietal Cognitive Control Network in Psychiatric Disorders: A Transdiagnostic Systematic Review of fMRI Studies

**DOI:** 10.3389/fpsyt.2016.00082

**Published:** 2016-05-18

**Authors:** Thérèse van Amelsvoort, Dennis Hernaus

**Affiliations:** ^1^Department of Psychiatry and Neuropsychology, South Limburg Mental Health Research and Teaching Network, EURON, School for Mental Health and NeuroScience MHeNS Maastricht University, Maastricht, Netherlands

**Keywords:** psychopharmacology, fMRI, psychiatric disorders, attention, cognition, prefrontal cortex, executive functioning, treatment

## Abstract

Executive function deficits, such as working memory, decision-making, and attention problems, are a common feature of several psychiatric disorders for which no satisfactory treatment exists. Here, we transdiagnostically investigate the effects of pharmacological interventions (other than methylphenidate) on the fronto-cingulo-parietal cognitive control network, in order to identify functional brain markers for future procognitive pharmacological interventions. Twenty-nine manuscripts investigated the effect of pharmacological treatment on executive function-related brain correlates in psychotic disorders (*n* = 11), depression (*n* = 4), bipolar disorder (*n* = 4), ADHD (*n* = 4), OCD (*n* = 2), smoking dependence (*n* = 2), alcohol dependence (*n* = 1), and pathological gambling (*n* = 1). In terms of impact on the fronto-cingulo-parietal network, the preliminary evidence for catechol-*O*-methyl-transferase inhibitors, nicotinic receptor agonists, and atomoxetine was relatively consistent, the data for atypical antipsychotics and anticonvulsants moderate, and interpretation of the data for antidepressants was hampered by the employed study designs. Increased activity in task-relevant areas and decreased activity in task-irrelevant areas were the most common transdiagnostic effects of pharmacological treatment. These markers showed good positive and moderate negative predictive value. It is concluded that fronto-cingulo-parietal activity changes can serve as a marker for future procognitive interventions. Future recommendations include the use of randomized double-blind designs and selective cholinergic and glutamatergic compounds.

## Introduction

How are pharmacological interventions for psychiatric disorders related to changes in functional brain correlates of executive functions? Impairments in executive functions, such as working memory, decision-making, planning, and attention, are a common feature of several psychiatric disorders. Extensive evidence in depression (1, 2), schizophrenia (SCZ) and psychosis (3, 4), bipolar disorder (5, 6), obsessive–compulsive disorder (OCD) (7, 8), substance dependence (9, 10), anxiety (11), autism spectrum disorders (12, 13), and attention-deficit/hyperactivity disorder (ADHD) (14, 15) suggests consistent impairments on a broad range of neuropsychological tests. For many of these disorders, there is compelling evidence that executive function deficits may be present before illness onset (16–18) and often persist beyond the acute phase of the disease (9, 19–21). With the exception of ADHD, there currently exists no successful pharmacological treatment for executive function deficits in psychiatric disorders.

The fact that assessments of executive functions in psychiatric disorders predict relapse/remission (9, 22, 23), functional outcome (24, 25), and in some cases, treatment response (26, 27) suggests that it is a core transdiagnostic symptom domain that requires adequate treatment. Recent reviews have suggested that increasing patient functioning and outcome may be achieved by boosting executive functions (28, 29), further increasing attractiveness of this symptom domain as a treatment target.

Then, do the available pharmacological interventions for the treatment of psychiatric disorders modulate executive function deficits? Based on the available literature, especially the dopamine- and noradrenaline-increasing effects of methylphenidate (MPH) have been consistently associated with normalizing effects in ADHD (30, 31) and procognitive effects in healthy volunteers (32). And, many other pharmacological agents with glutamatergic [e.g., memantine (33)], cholinergic [e.g., rivastigmine (33)], dopaminergic [e.g., modafinil (34) and atomoxetine (35)], and noradrenergic [e.g., atomoxetine (35)] properties have also been shown to modulate executive functions. Moreover, while not the *primary aim* of these agents, there is evidence for a modest improvement in some executive function domains for atypical antipsychotics (36), likely related to their dopamine- and serotonin-modulating properties.

It may be the case that pharmacological agents for psychiatric disorders, *which may or may not have the primary aim to modulate executive functions*, at least partly impact common substrates. Although executive functions are underlain by widely distributed networks, one brain network consistently associated with executive functions is the fronto-cingulo-parietal cognitive control network (37). Essential hubs in the fronto-cingulo-parietal network have consistently been associated with executive function domains such as the anterior cingulate cortex with cognitive control (38), dorsolateral prefrontal cortex (DLPFC) with working memory (37, 39), inferior frontal gyrus (IFG) and (pre-)supplementary motor area (SMA) with response inhibition (40–42), and the parietal lobules with visual attention and attention control (43, 44). As such, the fronto-cingulo-parietal network is ideally suited as a reference network, which can be used to evaluate the effect of pharmacological agents on executive function-related brain networks.

While especially the effect of MPH on cognitive control networks has been critically evaluated in relation to its pharmacological mechanism (30, 31), the aim of the *current review* was to systematically review the evidence for other available pharmacological interventions in a transdiagnostic fashion. This includes not only pharmacological agents with demonstrated procognitive effects but also agents that are not primarily used to ameliorate executive function deficits, such as antidepressants or antipsychotics. Relating plausible mechanisms of action to the effect of pharmacological interventions on functional brain correlates of executive functions could assist in further (I) validating the therapeutical effects of these agents and (II) elucidating brain mechanisms that could be targeted by future procognitive agents.

The use of functional magnetic resonance imaging (fMRI) to investigate brain correlates of executive functions is well established (45). More recently, this method has increasingly been used to evaluate the effects of pharmacological agents on brain function (46, 47). The strength of pharmacological fMRI is its ability to quantify activity changes in functional brain networks related to direct or downstream consequences of the pharmacological intervention. This enables the investigation of *common and distinct* drug effects on functional brain networks.

In this transdiagnostic systematic review, we aim to provide an overview of the effects of pharmacological interventions (other than MPH) on the fronto-cingulo-parietal cognitive control network in psychiatric disorders and to relate these to plausible neuropharmacological mechanisms. Using recent meta-analyses, we start off with a brief definition of the fronto-cingulo-parietal network. Next, we specifically evaluate original studies employing *strictly* executive functioning fMRI paradigms before treatment and after stable therapeutically efficacious dosing (monotherapy or adjunctive) had been implemented. We conclude with common transdiagnostic effects of pharmacological agents on the fronto-cingulo-parietal network, which could serve as markers for future procognitive interventions.

## Methods

PubMed was searched for studies published before October 23, 2015 using the initial Boolean phrase: (“fMRI” OR “functional magnetic resonance imaging”) AND (“cognition” OR “working memory” OR “attention” OR “decision-making” or “verbal learning” or “vigilance” or “processing speed” or “reasoning” or “problem solving” or “social cognition” or “verbal memory” or “visual learning” or “visual memory”) AND (“treatment”) AND (“pharmacology”). We followed up this initial search with a number of targeted searches in psychiatric disorders. To these aims, we replaced (“treatment”) AND (“pharmacology”) with (“pharmacology” OR “treatment”) AND (i) (“depression” OR “MDD”), (ii) (“schizophrenia” OR “psychosis”), (iii) (“bipolar” OR “mania” OR “cyclothymia” OR “rapid cycling”), (iv) (“substance dependence” OR “addiction” OR “substance abuse” OR “alcoholism”), (v) (“Tourette syndrome” OR “Tourette” OR “tic”), (vi) (“borderline” OR “personality disorder”), (vii) (“autism” OR “pervasive developmental disorder” OR “Asperger”), (viii) (“obsessive compulsive disorder” OR “OCD” OR “impulse control”), (ix) (“PTSD” or “post traumatic stress disorder”), and (x) (“anxiety” OR “fear” OR “phobia”). Titles and abstracts of all results were screened. Cross-referencing was performed on all included manuscripts and relevant reviews. Given the high number of recent meta-analyses and systematic reviews on the subject (30, 31, 48), we did not systematically review results of MPH treatment in psychiatric disorders. Treatments other than MPH in ADHD were included in the review if they met criteria.

Manuscripts were only considered if

(i) they were published in a peer-reviewed journal.
(ii) they were written in the English language.
(iii) they used the same fMRI paradigm at baseline and follow-up.
(iv) they reported group-level statistics; case studies were not included.
(v) pharmacological agents were specified, and results of the medicated group were reported (e.g., manuscripts combining samples of non-pharmacologically and pharmacologically treated patients were excluded).
(vi) the entire sample of patients was drug-free (in the case of monotherapy) or on stable monotherapy (in the case of adjunctive therapy) at baseline (washout allowed if necessary) and were stably and actively on (adjunctive) medication (no washout) during follow-up session(s). Concretely, “stably on medication” refers to repeated administration (>1; single dosing studies excluded) of the same efficacious drug dose.
(vii) they used fMRI paradigms that *only* measured aspects of executive functions. Tasks with stressful, painful, emotional, and/or rewarded components were excluded.
(viii) participants had a diagnosis of a psychiatric disorder according to DSM-IV criteria. Neurological disorders, such as stroke, dementia, and Parkinson’s disease, were excluded.

### Definition of and Rationale for the Cognitive Control Network as a Reference Network

The functional brain networks underlying higher cognitive and attention functions are widespread and complex with among others demonstrated cerebellar (49), occipital cortex (50, 51), striatal (52), and frontal cortical (39) involvement. In order to provide a clear delineation of the topic and facilitate the use of a reference network, we decided to review the effect of pharmacological agents on the fronto-cingulo-parietal cognitive control network. The cognitive control network has been hypothesized to play an essential role in orchestrating higher order behavior such as decision-making, action selection, and working memory (53). A comprehensive meta-analysis by Niendam et al. (37) demonstrated recurrent activity of a fronto-cingulo-parital cognitive control network during paradigms that assess working memory, response inhibition, behavioral flexibility, and other higher order skills (37). These results are in line with previous meta-analyses on functional brain networks underlying working memory, response inhibition, and selective attention, specifically and consistently revealing ACC, DLPFC, and superior and inferior parietal lobule (IPL) activity (41, 54–56). Therefore, the reported results in this manuscript (Table 1) include observed changes in parietal cortex (superior and IPL), insula, ACC, and frontal cortical areas, including DLPFC, ventrolateral PFC (VLPFC), and orbitofrontal cortex (OFC). In the discussion, these results will be linked to other notable findings of the available studies.

**Table 1:** **Overview of the effect of pharmacological agents on the fronto-cingulo-parietal cognitive control network**.

Reference	Diagnosis	*N* (treated)	*N* (PLC arm)	*N* (CTRL arm)	Study design	Treatment	Duration	Task	Main behavioral results	Main fMRI results	Timepoint and/or group comparisons	Task contrast
**Acetylcholinesterase inhibitors**												
Nahas et al. (58)	SCZ (atypical antipsychotics)	6	–	–	Randomized double-blind PLC-controlled crossover design	Donepezil adjunctive treatment	12 weeks	Covert verbal fluency task	No changes in task performance over time	On donepezil relative to PLC, 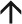 left IFG and left insula activity	Patients (donepezil vs. PLC)	Word generation vs. rest
Aasen et al. (59)^A^	SCZ (antipsychotics)	11	9	–	Randomized double-blind PLC-controlled trial	Rivastigmine adjunctive treatment	12 weeks	Number detection task	No (between-group) differences in task performance (over time)	–	–	–
Kumari et al. (60)^A^	SCZ (atypical antipsychotics)	11	10	–	Randomized double-blind PLC-controlled trial	Rivastigmine adjunctive treatment	12 weeks	*n*-back	No (between-group) differences in task performance (over time)	Over time and relative to PLC patients at T1, no 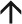 in right SFG activity	Rivastigmine (T0, T1) vs. PLC (T0, T1)	1-back vs. rest
**Anticonvulsants**												
Pavuluri et al. (61)	BD (pediatric; mania or mixed)	13	–	13; T0, T1	Open-label trial	Typical antipsychotics (weeks 1–4); lamotrigine (weeks 1–14)	14 weeks	Go/no go	Overall relative to CTRL, 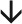 accuracy on No Go trials	Over time and relative to CTRL, greater 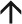 in left PFC and right MFG activity	Lamotrigine (T0–T1) vs. CTRL (T0–T1)	Go vs. No Go
									Over time and similar to CTRL, 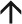 accuracy on Go trials	At T1 relative to CTRL, 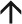 left M1* activity	Lamotrigine (T1) vs. CTRL (T0)	Go vs. No Go
Pavuluri et al. (62)^C^	BD (pediatric; mania or mixed)	11	–	14; T0, T1	Randomized double-blind trial	Divalproex	6 weeks	Go/no go	At T0 relative to CTRL, 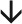 discrimination sensitivity; comparable to CTRL at T1^E^	Over time relative to CTRL, greater 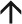 in left subgenual ACC and left insula FC in affective network	Divalproex (T0–T1) vs. CTRL (T0–T1)	During response inhibition
Schneider et al. (63)	BD (pediatric; mania or mixed)	10	–	9; T0	Open-label trial	Carbamazepine	8 weeks	CPT – identical pairs	At T0 and T1 relative to CTRL at T0, 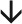 accuracy	Over time, 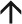 anterior PFC activity; n.s. at T1 relative to CTRL at T0	Carbamazepine (T0–T1); carbamazepine (T1) vs. CTRL (T0)	1-back vs. “1” detection
**Antidepressants: selective serotonin reuptake inhibitors and serotonin–noradrenaline reuptake inhibitors**												
Walsh et al. (64)	Major depression	20	–	20; T0–T2	Open-label trial	Fluoxetine	8 weeks	*n*-back	Over time relative to CTRL, 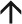 3-back accuracy; At T0–T2 relative to CTRL, 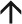 reaction times	–	–	–
Aizenstein et al. (65)	Late-life depression	13	–	13; T0	Open-label trial	Paroxetine	12 weeks	Spatial incompatibility task	At T1 relative to CTRL at T0, 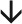 accuracy	Over time, 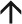 right DLPFC activity; at T1 relative to CTRL at T0, 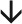 left DLPFC activity	Paroxetine (T0 vs. T1); paroxetine (T1) vs. CTRL (T0)	Contralateral vs. baseline, ipsilateral vs. baseline
Wagner et al. (66)^D^	Major depression	12	–	20; T0	Open-label trial	Citalopram	6 weeks	Stroop color-word task	Over time, greater 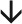 in reaction time for incongruent trials, relative to congruent trials^F^	Over time, 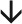 right VLPFC, SMA, IPL, and SPL activity	Citalopram (T0 vs. T1); citalopram (T0, T1) vs. reboxetine (T0, T1)	During incongruent condition
										At T1 relative to CTRL at T0, n.s. group differences^F^	Patients (T1) vs. CTRL (T0)	During incongruent condition
Gyurak et al. (67)	Major depression	79	–	34; T0, T1	Randomized open-label trial	Escitalopram (*n* = 26), Sertraline (*n* = 27), Venlafaxine ER (*n* = 26)	8 weeks	Go/No Go	–	Over time, remitters and CTRL show 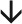 DLPFC activity; over time, no change in hypo-DLPFC activity of non-remitters	CTRL (T0 vs. T1); remitted (T0, T1) vs. non-remitted (T0, T1)	Go vs. No Go
										At T0 and T1, n.s. group differences in IPL activity between SSRI remitters and CTRL	Remitted (T0/T1) vs. CTRL (T0/T1)	Go vs. No Go
										At T0, 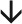 IPL activity in SNRI remitters than CTRL and SSRI remitters	Treatment (SSSRI, SNRI) vs. status (remitted, non-remitted, CTRL) vs. time (T0, T1)	Go vs. No Go
										At T1, 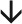 IPL activity in SNRI remitters than in CTRL		
van der Wee et al. (68)	OCD	14	–	–	Randomized double-blind trial	Paroxetine (*n* = 9), venlafaxine (*n* = 5)	12 weeks	*n*-back	Over time, responders, but not non-responders, 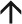 accuracy	Over time and relative to non-responders, 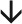 mean fronto-cingulo-parietal^G^ activity	Remitted (T0, T1) vs. non-remitted (T0, T1)	2-back vs. 0-back, 1-back vs. 0-back
Han et al. (69)	OCD	10	–	20; T0	Open-label trial	Escitalopram (*n* = 9), fluoxetine (*n* = 1)	16 weeks	Task-switching paradigm	At T0 and T1 relative to CTRL, 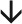 accuracy. Over time, 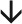 task-switching costs (RT)	Over time, 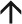 right ACC, right insula, right PCG, and right PC activity	Patients (T0 vs. T1)	Task switching vs. task-repeat
										At T1 relative to CTRL at T0, 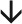 DLPFC^†^, DMPFC^†^, right MFC^†^ and PC^†^ activity, and 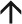 left insula* activity	Patients (T1) vs. CTRL (T0)	Task switching vs. task-repeat
**Atypical antipsychotics**												
Pavuluri et al. (62)^C^	BD (pediatric; mania or mixed)	11	–	14; T0 and T1	Randomized double-blind trial	Risperidone	6 weeks	Go/no go	At T0 relative to CTRL, 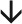 discrimination sensitivity; comparable to CTRL at T1^E^	Over time relative to CTRL, greater 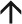 in insular FC in affective network	Risperidone (T0–T1) vs. CTRL (T0–T1)	During response inhibition
Schneider et al. (63)	BD (pediatric; mania or mixed)	10	7	10; T0	Randomized double-blind PLC-controlled trial	Ziprasidone	4 weeks	CPT – identical pairs	No between-group differences in task performance (over time)	Over time relative to PLC patients, greater 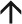 in right VLPFC/OFC activity	Ziprasidone (T0–T2) vs. PLC (T0–T2)	1-back vs. “1” detection
										At T1 and T2 compared to CTRL at T0, n.s. group differences in VLPFC/OFC activity	Patients (T0/T1/T2) vs. CTRL (T0)	1-back vs. “1” detection
Snitz et al. (70)	FEP	11	–	16; T0 and T1	Open-label trial	Risperidone (*n* = 7); olanzapine (*n* = 3); quetiapine (*n* = 1)	4 weeks	Spatial incompatibility task	At T0 and T1 relative to CTRL, 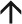 reaction times	Over time, 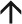 ACC, but not DLPFC, activity	Patients (T0 vs. T1)	Contralateral vs. baseline, ipsilateral vs. baseline
										At T1 relative to CTRL, n.s. group differences in DLPFC and ACC activity	Patients (T1) vs. CTRL (T1)	Contralateral vs. baseline, ipsilateral vs. baseline
Meisenzahl et al. (71)	SCZ	12	–	12; T0	Open-label trial	Quetiapine	12 weeks	*n*-back (degraded and non-degraded)	No (between-group) differences in task performance (over time)	Over time, 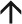 left VLPFC (non-degraded) and right precuneus (degraded) activity	Quetiapine (T0 vs. T1)	2-back vs. 0-back
Keedy et al. (72)	FEP	9	–	9; T0 and T1	Open-label trial	Risperidone (*n* = 6), ziprasidone (*n* = 1), haloperidol (*n* = 2)	4–6 weeks	Visual attention task	–	Over time, 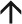 left FEF activity and 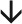 IPS and VMPFC activity; n.s. at T1 relative to CTRL	Patients (T0 vs. T1); Patients (T1) vs. CTRL (T1)	Prosaccade vs. fixation
										At T1 relative to CTRL, 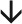 supramarginal gyri*, insula*, DLPFC*, SEF^†^ and ACC activity	Patients (T0 vs. T1); Patients (T1) vs. CTRL (T1)	Prosaccade vs. fixation
Ikuta et al. (74)	FEP	14	–	14; T0 and T1	Randomized double-blind trial	Risperidone or aripiprazole (*n* not specified)	12 weeks	Multi-source inference task	Over time, 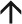 response accuracy; at T1 relative to CTRL, 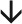 accuracy	Over time, 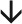 SFG activity; 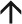 left SFG and rostral PFC activity	Patients (T0 vs. T1)	Interference vs. control
Keedy et al. (73)	FEP	14	–	12; T0 and T1	Open-label trial	Risperidone (*n* = 12), aripiprazole (*n* = 2)	4–6 weeks	Visual attention task	No (between-group) differences in prosaccade task performance (over time)	Over time, 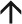 SEF, IPS and SPC activity; at T1 relative to CTRL, n.s. group differences	Patients (T0 vs. T1); Patients (T1) vs. CTRL (T1)	Prosaccade vs. fixation
									Over time, 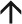 anticipatory saccades during predictive saccade task; at T1 relative to CTRL, n.s. differences	Over time, 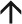 left IPS activity and 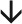 DLPFC activity; n.s. group differences	Patients (T0 vs. T1); Patients (T1) vs. CTRL (T1)	Predictive saccade vs. fixation
**Benzodiazepines**												
Wilcox et al. (75)	Alcohol use disorder; comorbid anxiety	7	–	9; T0	Open-label trial	Lorazepam plus disulfiram	5–7 days	Multimodal stroop task	Overall relative to CTRL at T0, 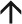 reaction times	Over time, 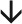 right IPL and insula deactivity	Lorazepam + disulfiram (T0 vs. T1)	Incongruent vs. congruent (Scan 1), incongruent + congruent (Scan 2)
**COMT inhibitors**												
Ashare et al. (76)	Smoking dependence (24-h abstinent)	20	–	–	Randomized double-blind PLC-controlled crossover design	Tolcapone	8 days	*n*-back	On tolcapone relative to PLC, 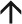 accuracy	On tolcapone relative to PLC, 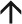 VMPFC deactivity	Patients (tolcapone vs. PLC)	0 + 1 + 2 + 3 back vs. baseline
									On tolcapone relative to PLC, 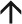 reaction times for Val/Val, 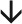 reaction times for Val/Met	On tolcapone relative to PLC, 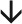 MFG activity and 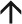 VMPFC deactivity in Val/Val and 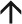 MFG and right DLPFC activity in Val/Met	Patients (tolcapone, PLC) for genotype (Val/Val, Val/Met)	0 + 1 + 2 + 3 back vs. baseline
Grant et al. (77)	Pathological gambling	12	–	12; T0	Open-label trial	Tolcapone	8 weeks	Tower of London task	No overall between-group differences in task performance	Over time, 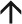 fronto-cingulo-parietal^H^ activity; at T1, fronto-cingulo-parietal^H^ activity normalized to CTRL at T0	Tolcapone (T0 vs. T1); tolcapone (T1) vs. CTRL (T0)	Planning condition vs. counting condition
**Nicotinic receptor agonists**												
Tregellas et al. (79)^B^	SCZ (non-smoking; antipsychotics)	16	–	–	Randomized double-blind PLC-controlled crossover design	Varenicline	4 weeks	Visual attention task	–	–	–	–
Tregellas et al. (80)^B^	SCZ (non-smoking; antipsychotics)	16	–	–	Randomized double-blind PLC-controlled crossover design	DMXB-A	4 weeks	Visual attention task	–	On DMXB-A 150 and 75 mg relative to PLC, 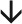 IPL and MFG DMN activity and 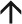 precuneus DMN activity	Patients (150 mg vs. PLC; 75 mg vs. PLC)	During prosaccade and rest
Loughead et al. (78)	Smoking dependence (72-h abstinent)	22	–	–	Randomized double-blind PLC-controlled crossover design	DMXB-A	13 days	*n*-back	On varenicline relative to PLC, 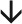 reaction times in highly dependent smokers, but not lowly dependent smokers	On varenicline relative to PLC, 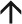 ACC and DLPFC activity; 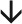 PFC activity (peak in right SFG and left IFG)	Patients (varencline vs. PLC)	3-back vs. 0-back, 2-back vs. 0-back, 1-back vs. 0-back
**Norepinephrine reuptake inhibitors**												
Schulz et al. (81)	ADHD (pediatric)	16	–	–	Randomized double-blind trial	Atomoxetine; MPH comparison (*n* = 18)	6–8 weeks	Go/No Go	Over time and similar to MPH, 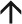 accuracy on No Go trials	Over time and similar to MPH, 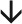 M1 activity	Atomoxetine (T0–T1) vs. methylphenidate (T0–T1)	Go vs. No Go
									Over time and similar to MPH, 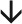 reaction time and reaction time variability on Go trials	Over time and relative to MPH, 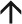 right IFG, left ACC, left SMA, and PCG activity (MPH 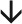 activity in these regions)	Atomoxetine (T0–T1) vs. methylphenidate (T0–T1)	Go vs. No Go
Bush et al. (82)	ADHD (adults)	11	10	–	Randomized trial	Atomoxetine; MPH comparison (*n* = 11)	6 weeks	Multi-source interference task	No differences in task performance (over time)	Over time, 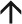 right DLPFC, left IPL, left SMA, and left insula activity	Atomoxetine (T0 vs. T1)	Interference vs. control
Chou et al. (83)	ADHD (pediatric)	22	–	20; T0	Randomized trial	Atomoxetine; MPH comparison (*n* = 20)	12 weeks	Stroop Counting Task	Over time and similar to MPH, 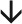 reaction time during incongruent trials	Over time relative to MPH, greater 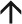 in left dorsal ACC and DLPFC activity and smaller 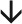 in left IFG activity	Atomoxetine (T0, T1) vs. methylphenidate (T0, T1)	Incongruent vs. congruent
Wagner et al. (66)^D^	Major depression	8	–	20; T0	Open-label trial	Reboxetine	6 weeks	Stroop color-word task	Over time, larger 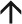 in reaction time for incongruent trials, relative to congruent trials^F^	–	–	–
Friedman et al. (84)	SCZ (atypical antipsychotics)	5	3	–	Randomized double-blind PLC-controlled design	Atomoxetine adjunctive treatment; PLC (*n* = 3)	8 weeks	*n*-back	–	At T1 relative to PLC patients, 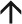 left DLPFC activity	Atomoxetine (T1) vs. PLC (T1)	2-back + 3-back vs. 0-back
**α2_A_ adrenergic receptor agonists**												
Bedard et al. (85)	ADHD (pediatric)	12	13	–	Randomized double-blind PLC-controlled trial	Guanfacine	6–8 weeks	Go/No Go	No differences in task performance (over time)	Over time relative to PLC patients, n.s. differences	Guanfacine (T0, T1) vs. PLC (T0, T1)	Go vs. No Go

*Appeared; not present at T0, present at T1.

*^†^Remained; present at T0 and T1*.

*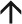, increased or higher; 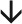, decreased or lower; T0, baseline; T1 and T2, follow-up; CTRL, healthy volunteers; PLC, placebo-treated; MPH, methylphenidate; FC, functional connectivity; DMN, default mode network. Psychiatric disorder: ADHD, attention-deficit/hyperactivity disorder; BD, bipolar disorder; OCD, obsessive–compulsive disorder; FEP, first-episode psychosis; SCZ, schizophrenia*.

*Brain regions: ACC, anterior cingulate cortex; DLPFC, dorsolateral prefrontal cortex; DMPFC, dorsomedial prefrontal cortex; FC, frontal cortex; IFG, inferior frontal gyrus; IPL, inferior parietal lobule; IPS, intraparietal sulcus; M1, primary motor area; MFC, medial frontal cortex; MFG, medial frontal gyrus; OFC, orbitofrontal cortex; PC, parietal cortex; PFC, prefrontal cortex; SEF, supplementary eye fields; SFG, superior frontal gyrus; SMA, supplementary motor area; SPL, superior parietal lobule; VLPFC, ventrolateral prefrontal cortex; VMPFC, ventromedial prefrontal cortex*.

^A,B^Identical sample. ^C,D^Part of same investigation. ^E,F^Significant effect/association in combined sample. ^G^Consists of ACC, DLPFC, premotor cortex, and PC. ^H^Consists of DLPFC, IPL, and frontopolar cortex.

## Results

The review was carried out according to PRISM (57) guidelines. The literature search in PubMed yielded 586 unique hits, of which 557 were excluded because they did *not* (i) utilize a pharmacological intervention or used MPH (38.2%), (ii) recruit patient populations (28.5%), (iii) employ a longitudinal fMRI design (17.8%), (iv) utilize a repeated dosing scheme (8.3%), (v) employ strictly cognitive tasks (4.4%), or (vi) were not written in the English language (2.8%). Thus, 29 manuscripts were included in this review, of which 26 used unique samples. In total, 431 individuals were scanned while medication free or on stable monotherapy at baseline and on monotherapy or adjunctive treatment at follow-up, in addition to placebo-treated patients and healthy volunteers.

Study designs, tasks, sample size, medication distribution, and main findings of the included studies are reported in Table 1. If available, results regarding (i) changes over time in patients vs. controls and (ii) patients vs. controls at follow-up were reported. Else, results regarding (i) change over time in patients and (ii) patients at follow-up vs. controls at follow-up, or (iii) patients at follow-up vs. controls at baseline were reported.

### Acetylcholinesterase Inhibitors

Three studies (two unique samples) investigated the effect of adjunctive actylcholinesterase inhibitors on executive function-related brain correlates before and after treatment. All studies were conducted in SCZ, and a total of 17 patients who were on stable antipsychotic medication were scanned before and after adjunctive treatment with actylcholinesterase inhibitors. Six patients were administered donepezil; the remaining 11 patients received rivastigmine.

#### Psychotic Disorders

None of the reported studies observed significant changes in task performance between placebo and medication sessions or between groups.

In a crossover design, Nahas et al. (58) investigated the effect of 12-week adjunctive donepezil treatment on verbal fluency-related brain activity. On donepezil relative to placebo, participants displayed increased left IFG and insula activity.

Aasen et al. (59) investigated brain activity during a number detection task in a 12-week rivastigmine trial. At follow-up and relative to placebo-treated patients, rivastigmine-treated patients did not show changes in fronto-cingulo-parietal activity. On the *n*-back task, which assesses selective attention and working memory, Kumari et al. (60) found a smaller increase in right superior frontal gyrus (SFG) activity from baseline to follow-up in the same sample of rivastigmine-treated patients, relative to placebo-treated patients.

### Anticonvulsants

We identified three manuscripts investigating the effect of anticonvulsants on the functional brain correlates of executive functions. All studies were conducted in pediatric bipolar disorder (age <18 years). A total of 34 pediatric patients were scanned before and after treatment. Thirteen patients were treated with lamotrigine, 11 with divalproex, and 10 with carbamazepine.

#### Bipolar Disorder

Pavuluri et al. (61) reported that, on a response inhibition task, pediatric bipolar patients displayed poorer overall (i.e., average of baseline and follow-up) accuracy on No-Go trials than healthy volunteers. However, similar to healthy volunteers, performance accuracy on Go trials increased from baseline to follow-up. From baseline to follow-up and compared to healthy volunteers, bipolar patients showed greater increases in left PFC and right medial frontal gyrus activity. At follow-up relative to healthy volunteers, increased left motor cortex activity appeared.

Another study by Pavuluri and colleagues (62) investigated the effect of divalproex on response inhibition and related functional connectivity. When divalproex-treated patients were combined with a parallel arm of risperidone-treated patients (see Atypical Antipsychotic treatment in Bipolar Disorder), discrimination sensitivity during a Go/No-Go task at baseline was poorer in patients than in healthy volunteers, but seemed to have normalized at follow-up. From baseline to follow-up and compared to healthy volunteers, divalproex-treated patients showed a greater increase in left subgenual ACC and left insula functional connectivity in an affective network. Directly comparing the divalproex- and risperidone-treated patient group, the latter group showed a trend-significant greater increase in left insula functional connectivity in the affective network.

Finally, carbamazepine-treated patients displayed poorer accuracy on a sustained attention task at baseline and follow-up compared to healthy volunteers at baseline (63). An increase in sustained attention-related anterior PFC from baseline to follow-up was observed. At follow-up, no significant differences were observed in anterior PFC activity compared to healthy volunteers at baseline.

### Antidepressants: Selective Serotonin Reuptake Inhibitors and Serotonin–Noradrenaline Reuptake Inhibitors

For treatment with selective serotonin reuptake inhibitors (SSRIs) and serotonin–noradrenaline reuptake inhibitors (SNRIs), six manuscripts were identified. Four studies were conducted in mood disorders, with a total of 124 patients scanned before and after treatment. Two studies were conducted in OCD, with a total of 24 patients scanned before and after treatment. Medication type distribution was as follows: 35 escitalopram (26 mood disorders), 31 venlafaxine (26 mood disorders), 27 sertraline (all mood disorders), 22 paroxetine (13 mood disorders), 21 fluoxetine (20 mood disorders), and 12 citalopram (all mood disorders).

#### Mood Disorders

On an *n*-back task, major depression patients compared to healthy volunteers had slower reaction times at baseline, which did not change after 2 and 8 weeks of SSRI treatment (64). However, compared to healthy volunteers, performance accuracy on the 3-back condition increased over time. Compared to healthy volunteers and over time, no activity changes in the fronto-cingulo-parietal cognitive control network were observed.

Aizenstein and colleagues (65) investigated the effect of SSRI treatment on task performance and brain activity during the preparing to overcome prepotency (spatial incompatibility) task, which assesses cognitive control. Compared to healthy volunteers at baseline, poorer performance accuracy at follow-up was observed in the sample of late-life depression patients treated with paroxetine. From baseline to follow-up, rule applying-related DLPFC, but not response overriding-related ACC, activity increased in patients. At follow-up, DLPFC activity was still lower than healthy volunteers at baseline.

Wagner et al. (66) investigated the effects of SSRI treatment on Stroop task performance and attention and interference-related brain activity. The decrease in response time between baseline and follow-up was greater for incongruent trials than for congruent trials when the SSRI-treated sample was combined with a sample receiving noradrenaline reuptake inhibitors (NRIs; see Noradrenaline Reuptake Inhibitors treatment in Major Depression). From baseline to follow-up, SSRI treatment was associated with widespread decreases in the fronto-cingulo-parietal network during the incongruent condition of the Stroop task (Table 1). When SSRI- and NRI-treated patients were combined, no differences in brain activity during the incongruent condition were observed at follow-up, relative to healthy volunteers.

In a large multicenter endeavor, healthy volunteers and remitted depression patients (treated with SSRIs or SNRIs) showed a decrease in response inhibition-related DLPFC activity from baseline to follow-up (67). Relative to healthy volunteers and remitters, non-remitters displayed DLPFC hypoactivity at baseline, which did not increase at follow-up. SSRI remitters and healthy volunteers showed a similar decrease in IPL activity from baseline to follow-up. At baseline, SNRI remitters compared to SSRI remitters and healthy volunteers displayed IPL hypoactivity, which did not increase to the level of healthy volunteers at follow-up.

#### Obsessive–Compulsive Disorder

Relative to non-responders, SSRI or SNRI treatment responders displayed increased performance accuracy on the *n*-back over time (68). From baseline to follow-up, responders relative to non-responders correspondingly displayed decreased activity in the fronto-cingulo-parietal network during the 1-back and 2-back condition.

Using a task-switching paradigm, Han et al. (69) showed that task-switching costs (task-switching reaction time − task-repeat reaction time) decreased in SSRI-treated patients from baseline to follow-up. However, patients displayed poorer accuracy at baseline and follow-up than healthy volunteers at baseline. From baseline to follow-up, task-switching activity increased in the fronto-cingulo-parietal network (Table 1). Compared to healthy volunteers at baseline, decreased frontal and parietal activity remained and increased insular activity appeared at follow-up (Table 1).

### Atypical Antipsychotics

For treatment with typical and atypical antipsychotics, seven manuscripts were identified. Two studies were conducted in bipolar disorder, with a total of 21 patients scanned before and after treatment. The remaining five studies were conducted in psychotic disorders, with a total of 60 patients scanned before and after treatment. The 36 patients received risperidone (11 bipolar), 11 ziprasidone (10 bipolar), 13 quetiapine, 3 olanzapine, 2 haloperidol, and 2 aripiprazole. Another 14 patients with psychotic disorder received either risperidone or aripiprazole, but numbers for each treatment were not reported.

#### Bipolar Disorder

In addition to divalproex (see Anticonvulsant treatment in Bipolar Disorder), Pavuluri and colleagues (62) also investigated the effect of risperidone on response inhibition-related functional connectivity in pediatric mania. From baseline to follow-up and compared to healthy volunteers, a greater increase in insular functional connectivity during response inhibition was observed in an affective functional connectivity network.

Schneider et al. (63) investigated the effect of ziprasidone on sustained attention in pediatric mixed/mania patients. Performance parameters on the continuous performance task (identical pairs version, similar to 1-back task) did not differ among treatment and control groups (over time). From baseline to follow-up and compared to a placebo-treated patient group, the zipradisone-treated group showed a larger increase in right sustained attention-related VLPFC and OFC activity. At days 7 and 28, no significant differences in sustained attention-related brain activity were observed between the entire patient sample (ziprasidone plus placebo) and healthy volunteers at baseline.

#### Psychotic Disorders

In another study using the preparing to overcome prepotency task, SCZ patients compared to healthy volunteers displayed increased reaction times at baseline and follow-up (70). From baseline to follow-up, response overriding-related ACC activity increased in SCZ patients, whereas rule applying-related DLPFC activity did not. At follow-up, task-induced ACC and DLPFC activity was comparable between SCZ patients and healthy volunteers.

In an open-label quetiapine trial, performance on the *n*-back task did not improve over time (71). However, task-induced left VLPFC and right precuneus activity increased in SCZ patients from baseline to follow-up.

Following treatment with atypical antipsychotics, Keedy et al. (72) reported activity normalization in anterior frontal and parietal areas during a visual attention task (Table 1). At follow-up, not all activity had normalized: compared to healthy volunteers, widespread reductions in fronto-cingulo-parietal activity had appeared or remained in SCZ patients (Table 1).

In a second study by Keedy et al. (73), patients and healthy volunteers did not differ in performance on the same visual attention task at baseline or follow-up. Activity in supplementary eye fields and parietal cortex normalized from baseline to follow-up (Table 1). During a presaccadic task, which probes frontostriatal motor and attention functions, increased left parietal and decreased DLPFC activity was observed from baseline to follow-up.

A final study using atypical antipsychotics in first-episode psychosis reported increased accuracy over time on an attention control task, which was still poorer than healthy volunteers at follow-up (74). In PFC, left SFG and anterior PFC activity increased from baseline to follow-up, although decreases in SFG activity were also reported.

### Benzodiazepines

We identified one manuscript using lorazepam plus disulfiram in substance dependence with comorbid anxiety disorder. A total of seven patients were scanned before and after treatment.

#### Alcohol Use Disorder

On a multisensory Stroop task, alcohol use disorder patients displayed overall slower reaction times than healthy volunteers at baseline (75). While at baseline, alcohol use disorder patients displayed attention and interference-related deactivity in parietal areas, there was slight activity at follow-up (i.e., decreased deactivity) (Table 1).

### Catechol-*O*-Methyl Transferase Inhibitors

For treatment with catechol-*O*-methyl transferase (COMT) inhibitors, two studies were identified. One study was carried out in smoking dependence; the other was carried out in pathological gambling. The 32 patients were scanned before and after tolcapone treatment.

#### Smoking Dependence

In a randomized double-blind placebo-controlled crossover design, Ashare et al. (76) observed that performance accuracy on the *n*-back task, relative to placebo, increased after 8 days of treatment with tolcapone. Relative to placebo, patients displayed increased ventromedial PFC (VMPFC) deactivity. On tolcapone relative to placebo, Val/Val carriers of the COMT gene displayed decreased medial PFC (MPFC) activity and increased (i.e., more) VMPFC deactivity during the task, while Val/Met carriers displayed increased VMPFC and right DLPFC activity.

#### Pathological Gambling

Pathological gambling patients and healthy volunteers showed similar overall accuracy on a Tower of London planning task (77). From baseline to follow-up, planning-related fronto-cingulo-parietal activity increased and seemingly normalized to that of healthy volunteers at baseline (Table 1).

### Nicotinic Receptor Agonists

We identified three papers (two unique samples) using nicotinic receptor agonists, with a total of 38 patients scanned before and after treatment. One study used α4β2 nicotinic receptor agonist varenicline in 22 smoking-dependent individuals. Another study used 3-(2,4-dimethoxybenzylidene)-anabaseine (DMXB-A), a partial α7 nicotinic agonist, as an adjunctive treatment in 16 non-smoking SCZ patients on stable antipsychotic treatment.

#### Smoking Dependence

Response times for correct trials on the *n*-back decreased on varenicline relative to placebo in highly dependent abstinent smokers, but not in their lowly dependent counterparts (78). Moreover, on varenicline relative to placebo, MPFC and DLPFC activity increased, specifically on the 2-back and 3-back level. In addition, whole brain analyses revealed frontal cortex activity decreases on varenicline relative to placebo, with peak deactivations detected in the right SFG and left IFG.

#### Psychotic Disorders

Relative to placebo, no changes in the fronto-cingulo-parietal network were observed during a visual attention task after a 1-month treatment with 75 and 150 mg of DMXB-A (79). However, differences in frontal and parietal default mode network activity were observed between the placebo and DMXB-A session (Table 1) (80).

### Noradrenaline Reuptake Inhibitors

For treatment with NRIs, five studies were identified. Three studies were conducted in ADHD (two pediatric and one adult sample), one in major depression, and one in SCZ (adjunctive treatment). A total of 64 patients were scanned before and after treatment, of which 56 received atomoxetine and 8 received reboxetine (all major depression).

#### Attention-Deficit/Hyperactivity Disorder

Similar to MPH-treated patients, accuracy increased on No-Go trials from baseline to follow-up in those receiving atomoxetine (81). Moreover, reaction times and reaction time variability decreased. From baseline to follow-up and similar to the MPH-treated group, primary motor cortex activity decreased during the response inhibition task. MPH and atomoxetine seemed to produce differential effects on the fronto-cingulo-parietal cognitive control network (Table 1): atomoxetine increased and MPH decreased activity from baseline to follow-up.

Bush et al. (82) reported that performance on an attention control task did not change over time or relative to placebo-treated patients. From baseline to follow-up, frontal cortical and parietal activity (Table 1) during an interference task increased in an atomoxetine-treated cohort of patients.

Similar to MPH-treated patients, reaction times on a Stroop task decreased over time in atomoxetine-reated patients (83). At brain level, differential effects of MPH and atomoxetine were observed. From baseline to follow-up and relative to MPH, greater increases in left DLPFC and ACC activity and a smaller increase in left IFG activity were reported.

#### Major Depression

In addition to SSRIs (see Antidepressant treatment in Mood Disorders), Wagner et al. (66) also investigated the effects of NRIs on response interference-related brain activity. Whereas SSRIs decreased fronto-cingulo-parietal network activity (Table 1), reboxetine did not.

#### Psychotic Disorders

Friedman et al. (84) investigated the effect of adjunctive atomoxetine pharmacotherapy on *n*-back-related brain activity in SCZ patients on stable treatment with atypical antipsychotics. Performance parameter changes were not observed among groups or over time. At follow-up relative to placebo-treated patients, increases in left DLPFC activity were observed.

### Noradrenergic Receptor Agonists

We identified one paper using α2_A_ noradrenergic receptor agonist guanfacine, investigating 12 pediatric ADHD patients before and after treatment.

#### Attention-Deficit/Hyperactivity Disorder

From baseline to follow-up and compared to placebo-treated patients, 6- to 8-week treatment with guanfacine was not associated with improved accuracy, reaction times, or response inhibition-related brain activity in the fronto-cingulo-parietal network (85).

## Discussion

The aim of this systematic review was to summarize the impact of pharmacological interventions for psychiatric disorders on the fronto-cingulo-parietal cognitive control network and to relate this to plausible neurochemical mechanisms of action. Here, we will first discuss the evidence per treatment class, followed by a review of transdiagnostic commonalities as potential markers for future procognitive treatments.

### Pharmacological Interventions, Their Effects on Functional Brain Correlates of Executive Functions and Neuropharmacological Mechanism

#### Cholinergic Targets

Despite the pivotal role of acetylcholine in executive functions (86, 87), few potentially procognitive cholinergic agents are currently available, with actylcholinesterase inhibitors and nicotinic receptor agonists used most frequently.

#### Acetylcholinesterase Inhibitors

With a placebo-controlled and placebo-controlled crossover design, the available preliminary evidence for adjunctive acetylcholinesterase inhibitors in SCZ was of good quality. In line with a large number of placebo-controlled clinical trials (88–92), adjunctive rivastigmine or donepezil treatment did not improve performance on attention and working memory tasks in SCZ. Moreover, in contrast to studies in Alzheimer’s disease (93, 94), two out of three manuscripts did not report task-related increases in fronto-cingulo-parietal activity (59, 60). Only Nahas et al. (58) observed increased IFG activity during a verbal fluency task after 12 weeks of treatment, a region also previously associated with verbal fluency (95) and likely associated with the Go/No-Go behavior required for this task. Kumari et al. (60) observed increased left SFG activity in placebo-treated patients over time, but not in the rivastigmine-treated patient group. These between-study differences may be explained by sample size (6 vs. 11 participants) or task demands, with verbal fluency tasks being dependent on lexical access speed and vocabulary size (96), in addition to attention and working memory.

There was, however, evidence for increases in occipital cortex and cerebellum activity (58, 59), previously reported to be related to visual attention, stimulus encoding, or motor speed (97, 98). Indeed, meta-analytic evidence suggests that acetylcholinesterase inhibitors may increase motor speed in SCZ (99), possibly explaining increased cerebellar activity.

The absence of any marked and consistent effects of acetylcholinesterase inhibitors on the fronto-cingulo-parietal cognitive control network in SCZ may be related to the suspected abnormalities in the cholinergic system. In SCZ, abnormalities have been observed at the receptor level, with evidence for a decrease in α7 nicotonic (100), β2 (101), and muscarinic receptors (102). Acetylcholinesterase inhibition, compared to direct targeting of receptors, may therefore not be the most efficient way to improve executive functions in SCZ. Second, tobacco desensitizes α7 and α4β2 nicotonic receptors (103, 104) and many participants in the available studies smoked tobacco. This could further decrease the procognitive potential of acetylcholinesterase inhibitors, although upregulated β2 receptors in smoking SCZ have also been associated with improved executive functions (105), requiring further investigation. Finally, all participants were already on atypical antipsychotics, which have been shown to increase frontal cortex acetylcholine release (106), fronto-cingulo-parietal activity (107, 108), and modestly improve performance on neuropsychological tests (36) (also see Atypical Antipsychotics), perhaps indicating a ceiling effect.

Thus, the current preliminary evidence suggests that adjunctive acetylcholinesterase inhibitors in SCZ do not markedly impact the fronto-cingulo-parietal cognitive control network, but may increase occipital cortex and cerebellum activity. Given the quality of the available evidence, these results likely reflect treatment effects, rather than practice or iatrogenic effects. In order to determine if the cholinergic system can be targeted to increase executive functions in SCZ, selective and direct targeting of nicotinic and muscarinic receptors may be a more fruitful strategy, although lack of specificity for the receptor subtypes has hampered the development of suitable drugs.

#### Nicotinic Receptor Agonists

The only available study for acetylcholine nicotinic receptor agonists in smoking dependence was a placebo-controlled crossover design, which reported decreased *n*-back reaction times in highly dependent smokers while on varenicline. Moreover, on varenicline relative to placebo, DLPFC and ACC activity increased in the entire sample of smoking-dependent individuals, while activity in other frontal cortical areas decreased. Modulation of these areas is in line with recent meta-analytic evidence and likely reflects varenicline’s ability to optimize activity of the cognitive control network while suppressing task-irrelevant activity of the default mode network (109). Behaviorally, these observations are in agreement with repeated dosing evidence showing that varenicline improves several aspects of executive functions in smokers (110–112) and non-smokers (113). Varencline’s ability to modulate fronto-cingulo-parietal activity is promising and eagerly awaits replication in other psychiatric disorders.

In non-smoking SCZ patients, partial α7 agonist DMXB-A was not associated with activity changes in the fronto-cingulo-parietal network during a visual attention task. However, task-related changes in hippocampal activity (79) and suppression of default mode network activity in frontal cortical and parietal areas (80) were observed. While no other data for SCZ were available, the trial evidence for DMXB-A (114, 115) and varenicline (116–120) in psychotic disorders has been mixed, perhaps related to underlying cholinergic abnormalities or smoking status. Here, the use of fMRI and neuropsychological tasks that emphasize working memory, response inhibition, and action selection abilities could be useful in assessing DMXB-A’s potential procognitive abilities in SCZ.

Varenicline is a nicotinic receptor partial agonist at α4β2 receptors and full agonist at α7 receptors, and DMXB-A is a partial agonist at α7 receptors. Both β2- and α7-selective compounds increase frontal cortex dopamine release in the rat (121), offering an explanation for varenicline and DMXB-A’s putative activity-modulating abilities in frontal cortical areas. Neuropharmacologically, α7 and α4β2 in frontal cortex (121), α4β2 receptors expressed on ventral tegmental area neurons (122) and α7 receptors expressed on glutamatergic projections to the ventral tegmental area (123) seem to underlie the increase in dopamine release.

#### Anticonvulsants

While anticonvulsants were originally developed to treat epileptic seizures, the majority of them have been shown to exert mood-stabilizing effects in bipolar (124, 125). Given that modulation of glutamatergic and γ-aminobutyric acid (GABA) activity is a common mechanism of these agents, and that excessive PFC glutamatergic signaling may contribute to cognitive impairments (126), it seems plausible to suggest that they could modulate executive functions.

With two open-label trials and one placebo-controlled study, the evidence for anticonvulsants in bipolar disorder was of moderate quality. In the open-label trials, there was no evidence for improvements in task performance (61, 63). This is in contrast to the only available placebo-controlled study, where divalproex treatment seemingly normalized response inhibition, but only when combined with a parallel group of risperidone-treated bipolar patients (62).

In the open-label trials, widespread normalization (increases) of anterior PFC and MPFC hypoactivity was observed after anticonvulsant treatment (61, 63). However, practice and placebo effects could not be excluded because of the lack of a placebo-controlled group and absence of prospective data for healthy volunteers (Table 1). In line with the findings of increased PFC activity in the open-label studies, subgenual ACC and insular functional connectivity increased during a Go/No-Go task in the placebo-controlled study (62). The regional specificity of these findings is noteworthy, given the consistent involvement of the ACC and insula in conflict monitoring and response inhibition (30). This may point toward treatment effects, but it is important that the findings for carbamazepine and lamotrigine are now replicated in placebo-controlled studies.

The inhibitory actions of lamotrigine, carbamazepine, and sodium valproate (127–130) at ion channels and *N*-methyl-d-aspartate (NMDA) receptors (131) are essential in modulating extracellular glutamate and GABA concentrations. Magnetic resonance spectroscopy studies in bipolar disorder have revealed alterations in frontal cortex, particularly ACC, glutamate, and glutamine concentrations (132, 133). Moreover, treatment with anticonvulsants (132), notably divalproex (134), alters VLPFC and ACC glutamate and glutamine levels, reflecting changes in glutamate concentrations, GABA concentrations, or both.

Summarizing, there is preliminary evidence that anticonvulsants impact, specifically increase, fronto-cingulo-parietal, especially ACC, activity in bipolar disorder, possibly reflecting changes in glutamate and/or GABA release at the neuronal level, and increased response inhibition at the behavioral level.

#### Antidepressants

Although impressive in total sample size, the quality of evidence for antidepressants in depression and OCD was suboptimal, with five out of six studies employing an open-label design. Moreover, four out of six studies only had access to healthy control data at baseline or did not utilize a control group (Table 1). This complicates interpretation of the findings and increases susceptibility to practice, placebo and, more generally, iatrogenic effects.

With the exception of one study reporting a greater increase in 3-back performance over time in patients vs. healthy volunteers (64), there was no evidence for improved task performance following antidepressant treatment in depression. These results are generally in line with clinical trials showing no marked effects of antidepressants on cognitive control in depression (135), a domain that all available studies in depression assessed. Nonetheless, they are puzzling, given the presence of response inhibition deficits in depression (136), the proposed role of serotonin in inhibitory control (40), and the observation that serotonergic manipulations modulate the cognitive control network in healthy volunteers (137–139).

Two studies in depression reported activity decreases over time, observing normalized DLPFC activity during a response inhibition task (67) and normalized frontal cortical and parietal activity during an interference/conflict resolution task (66) following antidepressant treatment. Moreover, there was an overall greater engagement of frontoparietal regions with increasing *n*-back difficulty in patients vs. healthy volunteers (64), consistent with the observation of DLPFC hyperactivity during the *n*-back task in untreated depression (140). Taken together, these data suggest that SSRIs decrease frontal cortical and parietal hyperactivity in treatment responders, in spite of any marked performance changes.

A modest increase in DLPFC activity was observed during a cognitive control task, which at follow-up was still lower than healthy volunteers at baseline (65). The reported increase in DLPFC activity could have been attenuated by practice effects, given that steady declines in task-related activity have been reported over time in healthy volunteers (64, 67) and the fact that this study only had access to healthy volunteer data at baseline. Moreover, the seemingly contrasting observations of increased and decreased PFC activity may also be related to diagnosis: DLPFC activity increases were observed in late-life depression, while studies reporting anterior PFC decreases were conducted in major depression (Table 1). A final explanation could be the significant variation in treatment duration, ranging from 6 to 12 weeks, which is especially noteworthy in light of the delayed actions of SSRIs (141, 142).

Serotonin–noradrenaline reuptake inhibitor treatment in depression did not seem to impact fronto-cingulo-parietal activity (67). This is in line with the only available study in depression directly comparing serotonergic and noradrenergic agents (66), where SSRIs were associated with decreased parahippocampal and amygdalar activity, and reboxetine with slightly increased activity in these regions (NRIs in other disorders discussed in Section “Noradrenaline Reuptake Inhibitors”). The differential effect of SSRIs, SNRIs, and NRIs on attention and cognition-related brain function may be related to regional variation in serotonin transporter (143), noradrenaline transporter (144), and serotonin receptor subtype 1_A_ (5HT1A) (145) expression. Still, they are surprising in light of the well-established modulating effects of NRIs on fronto-cingulo-parietal, specifically IFG, activity in ADHD (81, 83), and healthy volunteers (146).

All in all, the available results in depression suggest an effect of serotonergic treatment on the fronto-cingulo-parietal cognitive control network, but interpretation is severely hampered by a lack of randomized double-blind trials. In the absence of clear performance changes, we speculate that these activity changes could reflect improvement in the affective domain. Indeed, fronto-cingulo-parietal activity (changes) correlated with Hamilton Rating Scale for Depression scores (64, 66), and ACC metabolism predicts treatment response in depression (147).

The preliminary results in OCD were mixed on the level of performance and activity (changes), which could be related to study design (open label vs. double blind) or reference group (treatment responder vs. healthy volunteers at baseline) (68, 69). There were some indications of performance improvements, such as increased accuracy, in SSRI/SNRI treatment responders (68) and decreased reaction times from baseline to follow-up (69). However, comparison with a control group over multiple time points is essential to disentangle practice effects from treatment effects.

#### Atypical Antipsychotics

The quality of the evidence for atypical antipsychotics was moderate: three out of seven studies employed a randomized double-blind design, and six out of seven studies had baseline and follow-up data of a control group (Table 1).

Especially in psychotic disorders, atypical antipsychotics have been associated with modest improvements in executive function domains such as response inhibition, planning, and immediate recall (36, 148, 149). One out of two included studies in bipolar disorder reported increased task performance, with risperidone-treated patients showing comparable response inhibition to healthy volunteers but only when combined with a divalproex-treated group (also see Anticonvulsants) (62). In psychotic disorder, only performance on a visual attention task normalised (73) and two studies reported no performance differences at baseline and follow-up, relative to healthy volunteers (71, 72). Thus, there seems to be some overlap with clinical trials in terms of modestly improved executive functions on atypical antipsychotics.

Regardless of psychiatric disorder, the most consistent observation was increased, often normalized, anterior PFC (63, 70, 71) ACC (70), and parietal (73) activity following atypical antipsychotics treatment, the great majority being risperidone. Increased fronto-cingulo-parietal activity has also been observed when SCZ patients were switched from typical to atypical antipsychotics (107, 108) and from 4 to 8 weeks of treatment with olanzapine (150), all in all showing a consistent picture of increased fronto-cingulo-parietal activity on atypical antipsychotics. Outside of the fronto-cingulo-parietal network, decreases in ventral (74) and dorsal (72) striatal activity were observed in psychotic disorder. A correlation between caudate activity and risperidone dose (73) was also reported, consistent with the notion that antipsychotics decrease striatal dopaminergic hyperactivity.

Atypical antipsychotics are thought to increase frontal cortex DA release *via* 5HT1_A_ agonism (106, 151–153) and 5HT2_A_ antagonism (154, 155). In addition, increased frontal cortex acetylcholine and serotonin release has also been observed following administration of atypical antipsychotics (106, 151, 154). The observed increases in fronto-cingulo-parietal activity following atypical antipsychotics treatment may therefore be underlain by increased dopaminergic, serotonergic, and/or cholinergic activity in frontal cortex and their downstream effects. While the behavioral, neuroimaging, and neuropharmacological evidence seems to point toward modest procognitive effects of atypical antipsychotics, especially psychotic disorders are in need of replication with double-blind randomized designs to understand the exact extent of normalization within the fronto-cingulo-parietal network.

#### Benzodiazepines

With only one open-label study identified, the results for benzodiazepines were preliminary. There was no evidence for improved performance, and some hints at decreased parietal hyperactivity and decreased temporal hypoactivity at follow-up, although these activity changes might partly reflect practice effects.

#### Catechol-*O*-Methyl Transferase Inhibitors

A placebo-controlled crossover and open-label study were identified for COMT inhibitors, suggesting moderate quality of the available preliminary evidence.

One out of two studies reported performance changes; in smoking dependence, *n*-back performance accuracy increased on tolcapone, and there was an effect of COMT genotype on reaction times (76) (Table 1). Task-related activity changes were observed in both smoking dependence and pathological gambling, with the former showing increased VMPFC deactivity on tolcapone (76) and the latter normalized fronto-cingulo-parietal hypoactivity, albeit to activity of healthy volunteers at baseline and in the absence of a placebo-controlled group (77). These results are in line with tolcapone’s effect in healthy volunteers, where it has been reported to increase *n*-back performance (156) and optimize task-related brain activity (156, 157).

Although preliminary and modest in nature, the results for COMT are favorable and could suggest potential procognitive effects that remain to be replicated in large placebo-controlled clinical trials. Interestingly, tolcapone does not affect extracellular catecholamine concentrations when administered alone, but increases striatal and frontal cortex dopamine release after l-DOPA (158, 159) and clozapine (160) administration. This may suggest that the catecholamine-increasing effect of tolcapone on the fronto-cingulo-parietal network could be even greater in medicated individuals with Parkinson’s disease or psychotic disorder, opening up new avenues for adjunctive treatment.

#### Noradrenaline Reuptake Inhibitors

With three randomized studies (one reported to be double-blind), all having included a MPH-treated patient group and one additional placebo-treated patient group (Table 1), the evidence for atomoxetine in ADHD was of moderate to good quality. In line with clinical trial evidence (35), increased performance on response inhibition tasks was observed following atomoxetine treatment (81, 83).

Meta-analytical evidence in ADHD suggests that MPH among others consistently impacts IFG, dorsal ACC, and SMA activity (30), which were all areas consistently modulated by atomoxetine treatment (Table 1) and involved in conflict monitoring, response inhibition, and action selection (40). There were, however, differences between the two agents, such that atomoxetine and MPH exerted opposing effects on the same regions (81, 82) or that atomoxetine modulated task-relevant activity in one part of the fronto-cingulo-parietal network (e.g., ACC and DLPFC activity), while MPHs actions affected another part of the network (e.g., IFG) (83).

These results suggest that atomoxetine and MPH impact common substrates of the fronto-cingulo-parietal network, but also have a certain local and global task-dependent uniqueness. One tentative explanation could be that atomoxetine and MPH have almost comparable procognitive effects *via* differing underlying neurochemical mechanisms. MPH and atomoxetine’s dopamine- and noradrenaline-increasing properties (161) are for a major part directed to D1 and α2_A_ adrenergic receptors, the former involved in suppressing noise (162) and the latter in enhancing signal (163) of neuronal networks. Moreover, the atomoxetine-induced increase in noradrenaline levels in frontal cortex decreases after prolonged exposure, while this is not the case for MPH (164). The neuropharmacological properties of MPH and atomoxetine may differentially affect the reorganization of functional brain networks.

In contrast to amphetamine (165) and in line with previous work (166), adjunctive atomoxetine treatment in antipsychotics-treated SCZ patients did not improve task performance. Despite any performance changes, task-related activity increases in DLPFC were observed, as well as an increase in posterior cingulate gyrus activity. These are regions typically modulated by atomoxetine in ADHD (81). While preliminary, the combination of atypical antipsychotics and atomoxetine may have led to overstimulation of frontal cortex dopamine and noradrenaline release, thereby missing the narrow window in which frontal cortex dopamine and noradrenaline levels optimally mediate cognitive performance (163, 167). Replication with SCZ patients on typical antipsychotics or MPH as an adjunctive treatment may shed further light on these findings.

#### Noradrenergic Receptor Agonists

While there were no main effects of α2_A_ noradrenergic agonist guanfacine on response inhibition or associated brain function, this agent is clinically efficacious in treating symptoms of ADHD (168). Moreover, in smoking dependence, guanfacine modulates response inhibition and conflict resolution-related activity in among others SMA, DLPFC, VMPFC, and insula (169). In comparison to MPH and atomoxetine, guanfacine’s actions are confined to α2_A_ noradrenergic receptors, thereby specifically enhancing signal in working memory networks (163). Direct comparison of guanfacine with atomoxetine and MPH could be valuable in elucidating the degree to which the latter two compounds modulate fronto-cingulo-parietal activity *via* a signal-increasing mechanism.

### Common Transdiagnostic Changes in the Fronto-Cingulo-Parietal Network as Indicators of Successful Treatment

While executive function deficits are a universal symptom of psychiatric disorders, the underlying causes are not known. What is clear is that executive functions are underlain by an intricate and widely distributed network of brain regions. Theoretically, alterations in any of these regions can negatively affect network integrity, thereby giving rise to executive function deficits. As a result, pharmacological treatment of executive function deficits remains complex, reflected in the unsatisfactory treatment across psychiatric disorders. We identified common transdiagnostic changes in fronto-cingulo-parietal activity, which could serve as treatment markers.

Despite differences in experimental designs, treatment duration, and dosage, there were notable transdiagnostic effects of pharmacological agents on the fronto-cingulo-parietal network. In studies that also showed beneficial behavioral effects, or for which clinical trial data suggested beneficial effects, common activity changes in the fronto-cingulo-parietal network were (I) enhancement of activity in task-relevant areas and (II) suppression of activity in task-irrelevant areas.

In smoking dependence, tolcapone further suppressed activity in task-irrelevant areas such as the VMPFC (76), while in pathological gambling, it increased task-relevant fronto-cingulo-parietal activity (77). In line with the agent’s neuropharmacological action, the exact nature of activity changes was dependent on COMT genotype (Table 1). Similarly, nicotinic receptor agonist varenicline increased DLPFC and ACC activity in smoking dependence (78) and suppressed parietal and frontal cortical default mode network activity in SCZ (80). Atomoxetine, in most cases, increased task-relevant activity in DLPFC, ACC, IFG, and IPL in ADHD (81–83). Lastly, atypical antipsychotics increased DLPFC, ACC, and parietal cortex activity (70, 73), and decreased VMPFC activity (72). Overall, these results suggest positive predictive value of increased task-related fronto-cingulo-parietal activity as a marker of successful pharmacological treatment.

These were also transdiagnostic commonalities that displayed a degree of negative predictive value. Notably, increased task-relevant DLPFC activity was observed after adjunctive atomoxetine treatment in SCZ, together with increased task-irrelevant posterior cingulate activity, a region thought to be associated with the default mode network (80). Moreover, in depression, non-remitters showed task-relevant DLPFC hypoactivity (67). Finally, acetylcholinesterase inhibitors in SCZ did not consistently modulate fronto-cingulo-parietal network activity (58–60).

A final common observation was that the effects of pharmacological agents on the fronto-cingulo-parietal network were often lateralized. Unilateral effects could be related to lateralization of frontoparietal networks at rest (170). Indeed, changes in unilateral frontoparietal networks have also been observed following dopaminergic challenges (171). Lateralized functional networks could be the result of lateralized projections at the cellular level, which has been shown for among others dopamine (172), serotonin (173), and glutamate (174). Assessing laterality of the observed activity changes could be helpful in determining the specificity of treatment effects.

### Conclusion and Future Directions

The available evidence suggests that dopamine, noradrenaline, and acetylcholine agonists are most consistently associated with activity changes in the fronto-cingulo-parietal network, as measured with fMRI. Across disorders, increased activity in task-relevant areas or decreased activity in task-irrelevant areas were the most consistent findings, demonstrated by good positive and moderate negative predictive value. However, in order to fully assess the potential of these markers, more randomized double-blind studies are necessary.

The current review highlights the potential of a dimensional, transdiagnostic approach in future (pharmacological) neuroimaging studies, thereby paralleling a shift that is currently taking place in the field of clinical diagnostics and management. The search for procognitive agents has thus far been carried out within the context of categorical classifications, which has not lead to any major breakthroughs in the development of treatments.

A surprising observation was the lack of selective cholinergic, especially muscarinic, and glutamatergic, notably NMDA receptor, agents. Preclinical and proof-of-concept trial evidence have demonstrated promising procognitive potential for M1 allosteric modulators (86, 175) and selective NMDA antagonists (176). The lack of clinical trial and neuroimaging evidence may reflect unavailability of such compounds or potential safety issues of the available compounds (177). A recent initiative that may facilitate testing of novel compounds is the Medicine Chest initiative by the ECNP (www.ecnp.eu), which could aid clinical researchers in gaining access to new pharmacological tools.

## Author Contributions

DH and TA performed literature search, writing, and interpretation of the data.

## Conflict of Interest Statement

The authors declare that the research was conducted in the absence of any commercial or financial relationships that could be construed as a potential conflict of interest.
